# Brain alpha‐amylase: a novel energy regulator important in Alzheimer disease?

**DOI:** 10.1111/bpa.12597

**Published:** 2018-03-30

**Authors:** Elin Byman, Nina Schultz, Netherlands Brain Bank, Malin Fex, Malin Wennström

**Affiliations:** ^1^ Clinical Memory Research Unit, Department of Clinical Sciences Malmö Lund University Malmö Sweden; ^2^ Netherlands Institute for Neuroscience Amsterdam The Netherlands; ^3^ Unit for Molecular Metabolism, Lund University Diabetes Centre, Department of Clinical Sciences, Lund University Malmö Sweden

**Keywords:** Alzheimer's disease, α‐amylase, amyloid beta, astrocytes, dendritic spines, polyglucosan bodies

## Abstract

Reduced glucose metabolism and formation of polyglucosan bodies (PGB) are, beside amyloid beta plaques and neurofibrillary tangles, well‐known pathological findings associated with Alzheimer's disease (AD). Since both glucose availability and PGB are regulated by enzymatic degradation of glycogen, we hypothesize that dysfunctional glycogen degradation is a critical event in AD progression. We therefore investigated whether alpha (α)‐amylase, an enzyme known to efficiently degrade polysaccharides in the gastrointestinal tract, is expressed in the hippocampal CA1/subiculum and if the expression is altered in AD patients. Using immunohistochemical staining techniques, we show the presence of the α‐amylase isotypes AMY1A and AMY2A in neuronal dendritic spines, pericytes and astrocytes. Moreover, AD patients showed reduced gene expression of α‐amylase, but conversely increased protein levels of α‐amylase as well as increased activity of the enzyme compared with non‐demented controls. Lastly, we observed increased, albeit not significant, load of periodic acid‐Schiff positive PGB in the brain of AD patients, which correlated with increased α‐amylase activity. These findings show that α‐amylase is expressed and active in the human brain, and suggest the enzyme to be affected, alternatively play a role, in the neurodegenerative Alzheimer's disease pathology.

**Abbreviations**
Aβ40: amyloid beta 1–40
Aβ42: amyloid beta 1–42
AD: Alzheimer's disease
AMY: alpha (α)‐amylase
APP: amyloid beta precursor protein
CA1: cornus ammonis 1
GAA: acid alpha glycosidase
GP: glycogen phospholrylase
GDE: glycogen degrading enzyme
HB: Hirano bodies
IHC: immunohistochemistry
NC: non‐demented controls
NFT: neurofibrillary tangles
NFTL: neurofilament light chain
PGB: polyglucosan bodies
PMD: postmortem delay
SUB: subiculum
TLE: temporal lobe epilepsy

## Introduction

Alzheimer's disease (AD) is neuropathologically characterized by amyloid beta (Aβ)42 plaques and neurofibrillary tangles (NFT) 8. These hallmarks of AD are closely related to the synaptic/neuronal loss and cognitive decline 25 associated with the disease. However, studies using F^18^‐fluorodeoxyglucose (FDG)‐PET, have also shown a relation between Aβ plaque load and reduced glucose utilization in the AD brain 24, 31. The mechanism behind the low glucose utilization in AD patients is not fully understood and there is still some debate whether low glucose uptake is due to the AD associated neurodegeneration or if altered glucose metabolism precedes (or even causes) the neurodegeneration. Interestingly, studies on patients with mild cognitive impairment and/or risk of familial AD have shown that hypometabolism occurs before the neurodegeneration and atrophy 24, 27, 31, which favors the latter hypothesis. Hypometabolism has also been suggested to be related to downregulation of glucose transporters in AD patients 28. Other studies suggest brain insulin resistance, an event sometimes referred to as type 3 diabetes, as the potential culprit 41. In either case, it is clear that the research field is yet in its cradle and further studies are required to pinpoint the mechanisms underlying the altered glucose metabolism in the brains of AD patients. An interesting approach, so far not well explored, is to study factors involved in storage and degradation of energy reserves such as glycogen 13. Glycogen is a highly multibranched polysaccharide consisting of linear glucose chains linked together with alpha (α)^1^, ^2^, ^3^, ^4^ glyosidic bonds and chains branching off by α(1–6) glyosidic bonds 1, 11. It is synthesized by brain specific glycogen synthetase 34 and found foremost in the cytosol of astrocytes, but also in endothelial cells, pericytes and neurons 12. As much as 40% of the glucose in the brain is transformed into glycogen 44 and when there is an increased glucose demand, the cytosolic glycogen is degraded into glucose molecules again. The degradation is performed foremost by two enzymes; glycogen phosphorylase (GP), which phosphorylates single glucose molecules from the endpoint of the linear chain 1, 11, and glycogen debranching enzyme (GDE), which cleaves (hydrolyzes) the α(1–6) branching point 1. The capacity to produce and degrade glycogen has in recent years gained a lot of attention as astrocytes has been shown to convert glycogen into lactate, which in turn is exported into the energy demanding neurons. The energy reserve protects neurons against hypoxic stress 36 and plays a crucial role in memory formation 13, 17 and neurotransmitter production 44. Whether neurons themselves have a similar capability is still under debate.

The downside of the capability to synthesize and degrade glycogen becomes apparent when either of them are disturbed. If glycogen synthesis is increased or if degradation fails, polysaccharides can accumulate and form intracytoplasmic inclusions, termed polyglucosan bodies (PGBs) 13, 22. Examples of PGBs are corpura amylacea (CA) 16, multigranular glycogen 9, Lafora bodies 13 and amylose 22. The significance of PGBs is not fully understood, but they are often found in elderly or in patients suffering from different neurodegenerative diseases, such as AD, Parkinson's disease and epilepsy 5, 13, 40. PGBs are generally considered to be indicators of cell death and once formed they are difficult to degrade. The resistance to degradation is thought to be due to the fact that most of the PGB types (including amylose, CA and Lafora bodies) consist of less branched polysaccharides 22. Consequently, since GP only cleaves at the endpoint of the linear chains, the degradation is limited by the scarce access to endpoints in the unbranched polysaccharides. An enzyme capable of cleaving the α^1^, ^2^, ^3^, ^4^ bond within the linear chain (thereby enhancing the presence of endpoints) would therefore be beneficial. Such an enzyme, called acid alpha glycosidase (GAA), can be found within lysosomes in astrocytes and neurons 1, but whether other enzymes with similar properties are present in cells producing glycogen are still unknown. Interestingly, GAA belongs to a family of 30 enzymes called amylases and the most abundant and documented of these amylases is alpha (α)‐amylase. Just like GAA, α‐amylase hydrolyzes the α^1^, ^2^, ^3^, ^4^ bond and due to its capability to cleave multiple bonds within the linear chain it can very efficiently degrade glycogen 7. The α‐amylase is foremost present in the saliva (isoforms AMY1A, 1B and 1C) and in pancreatic juice (isoforms AMY2A and 2B), where it is responsible for converting starch, glycogen and other polysaccharides into smaller molecules susceptible for uptake in the intestinal lumen 45. The enzyme has also been found in other tissues such as the intestines, ovaries, liver, placenta, testis, lung and skeletal muscles 30, 46, indicating a role for α‐amylase also in these organs. No studies (to our knowledge) have demonstrated α‐amylase expression in the brain, but given the presence of polysaccharides and glycogen in the brain, it is not unlikely that also brain cells express this enzyme. Since the efficiency of glycogen degradation evidently is crucial for both glucose release and avoidance of PGB formation, it is clear that glycogenolysis plays a critical role in brain glucose metabolism. Hence, it is tempting to speculate that down‐regulated glycogenolysis could be important in AD neuropathogenesis. We therefore hypothesize that α‐amylase has a function in the brain and that it may be altered in response to AD pathology. To investigate this hypothesis, we used immunohistochemistry, immunoassays, activity assays and quantitative reverse transcription PCR (RT‐qPCR) to analyze the presence of α‐amylase in hippocampus of non‐demented individuals and patients with AD. We further analyzed the relationship between α‐amylase and Aβ, NFT and PGB accumulation.

## Material and Method

### Individuals included in the study

The study was performed on midlevel hippocampal samples from two cohorts. Cohort 1 includes (*n* = 3) non‐demented controls (NC), (*n* = 3), clinically and postmortem verified AD patients and (*n* = 1) AD patient with temporal lobe epilepsy (TLE) [Netherlands Brain Bank (NBB)]. Cohort 2 includes (*n* = 8) NC and (*n* = 12) AD patients (NBB). Demographics of individuals included in the study are given in Tables 1 and 2. Written informed consent for the use of brain tissue and clinical data for research purposes was obtained from all patients or their next of kin in accordance with the International Declaration of Helsinki. Medisch Ethische Toetsingscommissie (METc) of VU University has approved the procedures of brain tissue collection and the regional ethical review board in Lund has approved the study. Single nucleotide polymorphisms at position rs429358 and rs7412 of the *APOE* gene were determined by polymerase chain reactions using allele specific primers.

**Table 1:** Demographic data of cohort 1, qualitative assessment and cause of death of individuals included in the study.

Clinical Diagnosis	Gender (M/F)	Age (years)	APOE (genotype)	Neuropat. Ev. (NFT/Aβ/LB)	Cause of death
NC	F	72	3/3	1/A/0	Euthanasia, ovarian cancer
NC	F	75	2/3	1/A/0	Euthanasia, heart failure
NC	F	89	NA	1/A/0	Suicide
AD	M	82	3/4	5/C/0	Advanced Alzheimer's disease
AD	M	72	3/4	6/C/0	Pneumonia and stomach bleeding
AD	M	64	NA	6/C/6	Euthanasia, Alzheimer's disease
AD/TLE	F	90	NA	6/C/5	Dementia

Neuropat. Ev. = neuropathological evaluation; AD = Alzheimer's disease; NC = non‐demented controls; TLE = temporal lobe epilepsy; M = Male; F = Female; Aβ = amyloid beta; NFT = neurofibrillary tangles; LB = Lewy bodies; NA = not analyzed.

**Table 2:** Demographic data of cohort 2, qualitative assessment and cause of death of individuals included in the study.

Clinical diagnosis	Gender (M/F)	Age (years)	APOE (genotype)	Neuropat. Ev. (NFT/Aβ/LB) (Braak/ABC)	Cause of death
NC	M	70	3/2	1/O/3	Pneumonia, cardiogenic shock
NC	M	81	4/3	3/C/0	Pancreas carcinoma
NC	F	60	3/2	0/O/0	Metastasized mammacarcinoma
NC	F	92	3/4	3/O/1	Heart failure
NC	M	102	4/3	3/A/0	Ileus
NC	M	75	3/3	1/O/0	Cardiac arrest COPD
NC	M	79	3/2	1/O/0	Sepsis
NC	F	68	3/3	0/O/0	Euthanasia, Encephalitis
AD	F	83	3/3	4/C/6	Gastero‐enteritis, dementia
AD	F	88	3/3	5/C/5	Cachexia with vascular dementia
AD	F	65	4/3	5/C/0	Dementia
AD	F	88	3/4	5/C/0	Pneumonia
AD	M	85	3/3	4/C/0	Cardiac arrest
AD	F	96	3/3	4/B/0	Hearth failure, dementia.
AD	M	69	4/3	6/C/0	Pneumonia, subcortical dementia
AD	F	92	4/3	6/C/0	Atrioventricular block, AD
AD	F	70	4/4	6/C/0	Cachexia by dementia
AD	F	91	3/3	4/C/0	Cerebrovascular accident
AD	M	63	3/4	4/C/6	Metastases from unknown tumor
AD	F	78	3/4	6/C/0	Cachexia by dementia

Neuropat. Ev. = neuropathological evaluation; AD = Alzheimer's disease; NC = non‐demented controls; M = male; F = female; Aβ = amyloid beta; NFT = neurofibrillary tangles; LB = Lewy bodies.

### Brain sample preparation

#### Cohort 1

The hippocampal samples were directly after autopsy post‐fixed in paraformaldehyde (PFA) (4%) for 14–20 h and then incubated in phosphate buffered saline (PBS) with 30% sucrose for 4 days. The tissue was thereafter sectioned in 40 μm free floating sections and stored in −20°C until used for immunohistochemistry.

#### Cohort 2

Each hippocampal sample was fresh frozen at autopsy and the frozen samples were divided into two 3 mm thick sections. One of the two sections (from each individual) was incubated in PFA (4%) for 4 h, incubated in 30% sucrose for 3 days and thereafter sectioned in 40 μm free floating sections. The other section was kept frozen for mRNA purification (RT‐qPCR) and homogenization. Prior to analysis the cornus ammonis 1 (CA1) and subiculum brain area of each hippocampal section was divided into three samples containing (i) CA1, (ii) the intermediate of CA1 and (iii) Subiculum (CA/SUB) and Subiculum (SUB).

### Immunostaining procedures and analysis of IHC intensity

The PFA fixed brain sample sections from cohort 1 were immunohistochemically (IHC) stained with an antibody directed against α‐amylase 1A (AMY1A) (rabbit‐anti‐AMY1A; Thermo Fisher Scientific, Waltham, MA, USA) or α‐amylase 2A (AMY2A) (rabbit‐anti‐AMY2A; Thermo Fisher Scientific, Waltham, MA, USA). Briefly described, tissue sections were quenched in 3% H_2_O_2_ and 10% methanol for 30 minutes and incubated with Impress reagent kit blocking solution (Vector Laboratories, Burlingame, CA, USA) for 1 h at RT, followed by incubation with rabbit‐anti‐AMY1A or rabbit‐anti‐AMY2A in blocking solution overnight at 4°C. Sections were thereafter incubated with anti‐rabbit Igs Impress reagent kit secondary antibodies (Vector Laboratories, Burlingame, CA, USA) for 2 h at RT followed by peroxidase detection for 3 minutes (0.25 mg/mL diaminobenzidine and 0.012% H_2_O_2_).

Cellular location of AMY1A or AMY2A was analyzed by sequential double immunofluorescence staining using Impress excel R.T.U antibody kit (Vector Laboratories, Burlingame, CA, USA). Antibodies directed against either the astrocyte marker glial fibrillary acidic protein (GFAP) (mouse‐anti‐GFAP; Dako, Glostrup, Denmark), neurofilament light chain (NFTL) (mouse‐anti‐neurofilament‐L, Thermo Fischer Scientific, Waltham, MA, USA), Aβ1–16 (mouse‐anti‐Aβ_1–16_, clone 6E10; Covance, Princeton, NJ) or p‐tau (rabbit‐anti‐Thr 181; Santa Cruz Biotechnology, Dallas, TX, USA) in combination with the AMY1A or AMY2A antibody. The sections were incubated in blocking solution (2.5% normal horse serum in KPBS) for 1 h at RT, followed by incubation with primary antibodies (mouse‐anti‐GFAP, mouse‐anti‐Aβ1–16 or rabbit‐anti‐p‐tau) in blocking solution overnight at 4°C. Sections were thereafter incubated with appropriate (ie, either goat‐anti‐rabbit Igs or goat‐anti‐mouse Igs) amplifier antibody, for 2 h at RT followed by incubation with Dylight 488 conjugated horse‐anti‐goat antibody (Vector Laboratories, Burlingame, CA, USA) for 2 h at RT. The sections were then incubated with blocking solution [5% goat serum (Jackson Immunoresearch, Westgrove, PA, USA) in KPBS] for 1 h at RT followed by incubation with AMY1A or AMY2A antibody in blocking solution overnight at 4°C. Finally, sections were incubated with goat‐anti‐rabbit Dylight 594 (Invitrogen, Carlsbad, CA, USA) for 2 h at RT. Cellular location of AMY1A positive dendrites/DS (AMY1A + DS) were analyzed by double staining, where the sections were incubated in blocking solution (5% goat serum in KPBS), containing AMY1A antibody and NFTL antibody, for 48 h in 4°C. The sections were thereafter incubated with goat‐anti‐rabbit Dylight 594 and goat anti‐mouse Alexa 488 (Thermo Fischer Scientific, Waltham, MA, USA). All sections were mounted with Vectashield Set mounting medium containing DAPI (Vector Laboratories, Burlingame, CA, USA). Co‐localization between AMY1A and Aβ1‐16, p‐tau and NFTL and between AMY2A and Aβ1‐16, GFAP was confirmed by the use of Olympus AX70 light microscope equipped with 20× objective and confocal microscopy 63× objective (Ziess LSM 510, Ziess, Oberkochen, Germany) and Zen software. To verify the specificity of the AMY1A antibody, we also stained the tissue with an antibody directed against the full‐length native salivary α‐amylase (sheep‐anti‐salivary α‐amylase; Abcam, Cambridge, UK) (nAMY1A) and the full‐length native pancreatic α‐amylase (sheep‐anti‐pancreatic α‐amylase; Abcam, Cambridge, UK) (nAMY2A). In short, the sections were quenched, incubated in blocking solution [5% donkey serum (Sigma Aldrich, St. Louis, MO, USA)] followed by an incubation overnight in 4°C in the primary antibody. The next day the section were incubated in HPR conjugated secondary (donkey anti sheep; Abcam, Cambridge, UK) for 2 h at RT followed by peroxidase detection for 3 minutes. The specificity of the AMY1A antibody and AMY2A antibody was further analyzed using Western blot. Hippocampal tissue from a NC and an AD patient was homogenized in lysate buffer and separated together with α‐amylase controls [porcine pancreatic α‐amylase (Sigma Aldrich, St. Louis, MO, USA) and human salivary α‐amylase (Sigma Aldrich, St. Louis, MO, USA)] on 10% SDS‐page gel. The proteins were thereafter transferred to a PVDF membranes (BioRad, Hercules, CA, USA) and incubated with blocking solution (5% milk powder in PBS‐Tween) for 1 h followed by incubation with AMY1A or AMY2A in blocking solution. The following day the membranes were incubated with anti‐rabbit HRP (1:10 000, Abcam, Cambridge, UK) for 1 h at room temperature. Luminata Forte Western HRP Substrate (Millipore, Darmstadt, Germany), ChemiDoc XRS + System (BioRad, Hercules, CA, USA) and Image Lab Software (BioRad, Hercules, CA, USA) were used to visualize the protein bands.

### mRNA purification and RT‐qPCR

The frozen CA1 samples (*n* = 20) and Subiculum (SUB) samples (*n* = 19) from cohort 2 (∼80 mg) from each individual were homogenized in gentleMACS M tubes (Miltenyi Biotec, Bergisch Gladbach, Germany) with 900 μL Qiazol lysis buffer (Qiagen, Venlo, the Netherlands). Total RNA was purified using RNeasy Plus Universal Mini Kit (Qiagen, Venlo, the Netherlands) according to the manufacturer's instructions. Concentration and purity of the RNA was quantified using Take 3 and Eon (Biotek, Winooski, VT, USA) and all samples were diluted in RNase free water to obtain the same RNA concentration. Thereafter, the mRNA was subjected to reverse transcription by using Maxima first strand cDNA synthesis kit (Life Tech, Carlsbad, CA, USA) according to manufacturer's instructions and the prepared cDNA was then mixed with Maxima probe/ROX QPCR mastermix (Life Tech, Carlsbad, CA, USA) together with probes for α‐amylase (HS00420710_g1) (capturing AMY2A, AMY1A, AMY1C, AMY1B and AMY2B) as well as the housekeeping genes ribosomal protein L13A (RPL13A) (HS04194366_g1) and hydroxymethylbilane synthase (HMBS) (Hs00609296_g1) (Applied Biosystems, Foster City, CA, USA). The RT‐qPCR reactions were carried out using Viia 7 system (Applied Biosystems, Foster City, CA, USA) and the relative expression in mRNA level was calculated using the 2‐^ΔCt^ method 38 and normalized against the geometric mean of the two housekeeping genes RPL13A and HMBS.

### Alpha‐amylase sandwich ELISA

The presence of α‐amylase in the homogenates of CA1/SUB in cohort 2 (*n* = 20) was measured using Human AMY1A ELISA kit (Nordic BioSite, Täby, Sweden), according to the manufacturer's protocol. Notably, the antibodies used in this kit are unable, according to the manufacturer, to distinguish between AMY2A and AMY1A and this assay therefore detects both AMY2A and AMY1A. The α‐amylase concentration was calculated for each sample and normalized with total protein content given by bicinchoninic acid assay (BCA) (Thermo Fischer Scientific, Waltham, MA, USA).

### Alpha‐amylase activity assay

The activity of α‐amylase in hippocampal homogenates from each individual in cohort 2 (*n* = 19) was analyzed using Amylase assay kit colorimetric (Abcam, Cambridge, UK) according to manufacturer's protocol. A kinetic measurement was done and the absorbance at 405 nm (OD_405nm_) increase/decrease over 30 minutes (ΔT^30^) were calculated for each sample. Analysis of one AD patient yielded an activity ten times higher than the activity found in the other individuals and was therefore considered as an outlier and consequently removed from the analysis.

### Periodic acid Schiff staining

PFA fixed brain sections from both cohort 1 and 2 were selected and mounted on glass‐slides and left to dry. The sections were rinsed in deionized water and treated with periodic acid from Periodic acid Schiff (PAS) kit (Sigma Aldrich, St. Louis, MO, USA) for 5 minutes and rinsed with deionized water. Thereafter sections were treated with saturated dimedone solution (Sigma Aldrich, St. Louis, MO, USA) for 20 minutes in 45°C, followed by three rinses with deionized water and incubation with Schiff base solution (Sigma Aldrich, St. Louis, MO, USA) for 13 minutes. Finally, sections were washed in tap water for 2 minutes and mounted with DPX (Sigma Aldrich, St. Louis, MO, USA). The sections from cohort 2 were analyzed with Olympus AX70 (40× objectives) and the PAS positive areas in two pictures captured from three sections from each individual were analyzed using ImageJ. The presence of PAS in the samples is presented as the mean ratio of PAS positive area per total area.

### Analysis of amyloid beta levels

The frozen CA1/SUB samples from cohort 2 (∼80 mg) (*n* = 19) from each individual were added to 600 μl lysis buffer containing HEPES and detergent IGEPAL (Abcam, Cambridge, UK) and homogenized using a syringe. The homogenates were thereafter centrifuged and the supernatants were collected. Levels of Aβ40 and Aβ42 in the supernatants were measured using the V‐PLEX Plus Aβ Peptide Panel 1 (6E10) kit for measuring Aβ38, Aβ40, Aβ42 (Mesocale Discovery, Rockville, MD, USA). The levels of Aβ38 in the vast majority of the samples (more than 75%) were below detection level and thus values of Aβ38 will not be described. Readings of duplicate standards and duplicate samples were averaged and Aβ concentrations determined by interpolation of a 4‐parametric curve fit. The ratio of Aβ42 and Aβ40 (Aβ42/Aβ40) for each individual was also calculated.

### Statistical analysis

Statistical analysis was performed using SPSS software (version 24 for Mac, SPSS Inc, Chicago, IL, USA). Kolmogorov–Smirnov test was used to verify normal distribution. Normally distributed samples (α‐amylase expression in CA1 and α‐amylase levels) were analyzed using student *t*‐test. Non‐normally distributed samples (α‐amylase expression in SUB, α‐amylase activity, Aβ42, Aβ40 and ratio of Aβ42/40) were analyzed using non‐parametric Mann–Whitney test. None of the measured variables correlated with increased age. As we found a negative correlation between PAS positive area and postmortem delay (PMD), differences in PAS positive area were analyzed using ANOVA with PMD as covariate. Correlations between PAS and α‐amylase expression in SUB as well as α‐amylase activity were analyzed using partial correlation with PMD as covariate. All other correlation analyses were analyzed with Spearman correlation test. Results are presented as mean ± standard deviation. Values of *P <* 0.05 were considered statistically significant, *P*‐values between 0.05 and 0.1 were considered to be a trend towards significance.

## Results

### Alpha‐amylase 1A and 2A show cell specific immunoreactivity in human brain

**Figure 1:**
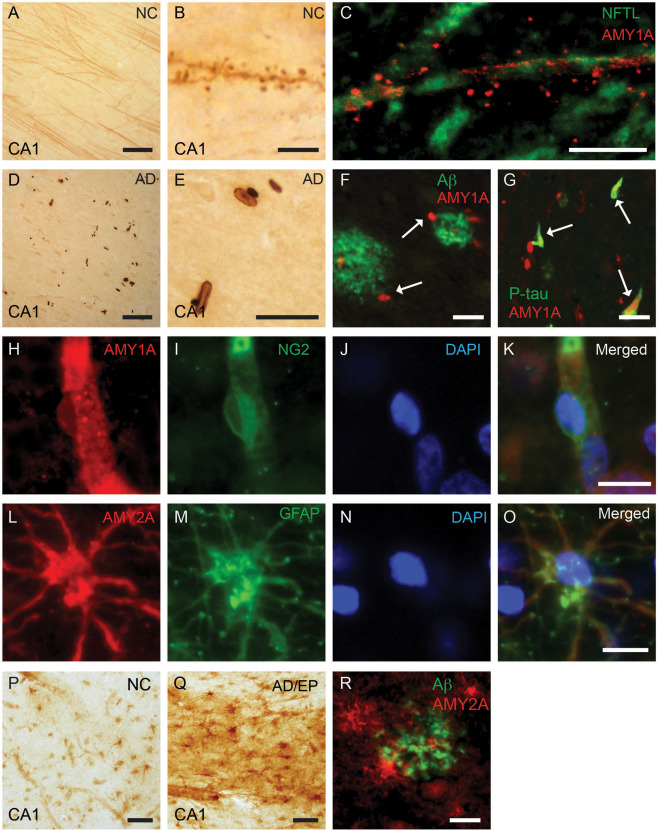
Immunostainings and cellular localization of alpha (α)‐amylase 1A and 2A in hippocampus of human postmortem brain tissue. Image in (**A**) shows an immunohistochemical (IHC) staining of α‐amylase 1A (AMY)1A revealing AMY1A positive dendrite‐like processes in Cornu Ammonis (CA1) of a NC (brown). A higher magnification of the same staining is found in (**B**), visualizing dendritic spine protrusions aligning the dendritic like projections. Image in (**C**) demonstrates the association between AMY1A (red) and NFTL (green) in CA1. Image in (**D**) shows AMY1A positive inclusions (brown) resembling HB in CA1 of an AD patient. The higher magnification of CA1 seen in image (**E**) demonstrates the morphology of the HB like inclusion bodies (brown). Double immunofluorescence staining in image (**F**) shows AMY1A + HB (red) adjacent to amyloid beta (Aβ) plaques (green). Double immunofluorescence staining of AMY1A and p‐tau seen in image (**G**) demonstrates that AMY1A + HBs (red) and p‐tau positive tangles (green) are occasionally co‐localized (orange). Images in (**H–K**) show that AMY1A (red in H), NG2 (green in I) and DAPI (blue in N) co‐localize (merged in K) indicating that the AMY1A + cells adjacent to vessels are pericytes. Images in (**L–O**) show double immunofluorescent staining of astrocyte marker GFAP (L), AMY2A (M) and DAPI (N) and co‐localization (merged in O) indicated that the AMY2A positive glial cells are astrocytes. Image in (**P and Q**) show a IHC staining of AMY2A positive astrocytes (brown) in CA1 from one NC (P) and the AD patients with TLE (Q). The AD with TLE patient (Q) display displayed high numbers of strongly stained AMY2A positive astrocytes (brown). Double immunofluorescent staining of AMY2A (red) and Aβ (green) in AD patient show AMY2A positive astrocytes associated with Aβ plaque (**R**). Scale bar in (A, D, Q, R) = 50 µm. Scale bar in (B, C, H–O) = 10 µm. Scale bar in (E, F, G, P) = 25 µm.

To investigate the potential presence and cellular localization of α‐amylase in the human brain, we initiated our studies by immunohistologically stain PFA fixed hippocampal/enthorhinal cortex (EC) sections (cohort 1) against AMY1A and AMY2A. The staining showed that AMY1A in cornu ammonis 1 (CA1) was associated with cellular structures resembling dendrites and dendritic spines (DS) (Figure 1A), but rarely neuronal cell bodies. These long projections were aligned with protrusions/grains (Figure 1B). To confirm that the AMY1A staining was associated with neuronal processes, we double stained the sections against AMY1A and the NFTL. Analysis using confocal microscopy showed that AMY1A positive DS like processes were closely associated with NFTL positive neuronal projections (Figure 1C). The AMY1A positive DS like processes, heron called DS, were clearly distinguishable in NC (Figure 1A), but to a large extent lost in AD patients (Figure 1D). The AMY1A staining also revealed oval and rod‐like inclusions sharing a morphology with Hirano bodies (HB) 29, 39 (hereon therefore called HB) (Figure 1D and E). The HB were seen both in CA1 (Figure 1D) and ML of both NC and AD patients, but to a larger extent in AD patients (Figure 1A and D). Further double immunofluorescence staining showed that many of the AMY1A positive HB in AD patients were associated with plaques positive for Aβ (Figure 1F) and co‐localized with p‐tau positive tangles (Figure 1G). Finally, we also noted AMY1A positive cells associated with vessels in both NC and AD group. These cells resembled pericyte cell bodies, which was confirmed by a double immunofluorescence staining against the pericyte marker neural‐glial antigen 2 (NG2) and AMY1A (Figure 1H–K). To verify the specificity of the antibody, we immunostained the CA1 with an additional AMY1A antibody, which is directed against the full‐length human salivary α‐amylase (nAMY1A). Although the staining yielded a higher background with less distinct spines, the nAMY1A stained dendritic like projections, HB‐like inclusions and vessel‐associated cell bodies resembling pericytes (Supporting Information Figure S1A–C). We also analyzed the AMY1A specificity with Western blot. Wells loaded with standard human salivary α‐amylase and hippocampal NC homogenates showed bands at approximately 50–52 kDa (Supporting Information Figure S1D). Two additional band around 40 kDa appeared in the well loaded with homogenates (Supporting Information Figure S1D), which most probably corresponds to cleavage product known to appear in response to protease cleavage 14.

Staining against AMY2A revealed a different staining pattern compared with AMY1A, where foremost cells with glial morphology were stained. Double immunofluorescence staining showed that the AMY2A positive glial cells were also positive for the astrocyte marker GFAP (Figure 1L–O). Immunohistochemistry staining of AMY2A showed that AMY2A positive astrocytes were found in both NC and AD patients (Figure 1P). The immunoreactivity in the AD patient with TLE was particularly distinct, displaying high numbers of strongly stained AMY2A positive astrocytes in both hippocampus (Figure 1Q) and entorhinal cortex. Further double immunofluorescence staining of AMY2A and Aβ revealed that AMY2A positive astrocytes were occasionally associated with plaques positive for Aβ in hippocampus of AD brain (Figure 1R). Similar staining pattern were observed after staining against the full‐length human pancreatic α‐amylase (nAMY2A), that is, AMY2A positive glial cells were noted in both NC and AD, but scattered and strongly immunoreactive nAMY2A glial cells were particularly noted in AD patients. The AD/TLE patient displayed an overall increased AMY2A immunoreactivity (Supporting Information Figure S1E–G). Western blot analysis of the AMY2A antibody revealed bands at 50–52 kDa in wells loaded with pancreatic porcine α‐amylase and hippocampal AD homogenates (Supporting Information Figure S1H). Two additional bands, located between 40 and 50 kDa, were detected in the latter well (Supporting Information Figure S1H), which corresponds to known protease cleavage products 14.

### Alpha‐amylase gene expression is decreased in Alzheimer's disease patients

**Figure 2:**
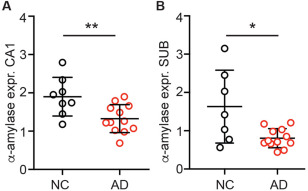
Relative alpha (α)‐amylase expression normalized against values of housekeeping genes ribosomal protein L13A (RPL13A) and hydroxymethylbilane synthase (HMBS) from NC and AD. Column scatter plots in (**A**) show the significantly lower expression of α‐amylase in cornus ammonis 1 (CA1) in AD compared with NC. Column scatter plots in (**B**) show the significantly lower expression of α‐amylase in Subiculum (SUB) in AD compared with NC. Comparison analyses are performed using student *t*‐test and non‐parametric Mann–Whitney. * indicates *P <* 0.05 ** indicates *P <* 0.01.

To investigate α‐amylase gene expression in the brain and whether this potential expression is altered in brain areas affected by AD pathology, we performed RT‐qPCR analyses on samples from two adjacent brain areas, the CA1 and SUB (cohort 2). Both these hippocampal regions are known to contain AD pathology, that is, Aβ plaques and NFT, in later stages of AD 10. The analysis of the samples from both areas revealed significant lower relative expression of α‐amylase normalized to housekeeping genes RPL13A and HMBS in AD patients compared with NC (Figure 2A and B). The α‐amylase expression in APOE ɛ4 carriers vs. APOE ɛ4 non‐carriers did not differ regardless of the areas analyzed or clinical diagnosis.

### Alpha‐amylase gene expression correlates with neuropathological evaluation and amyloid beta levels

**Figure 3:**
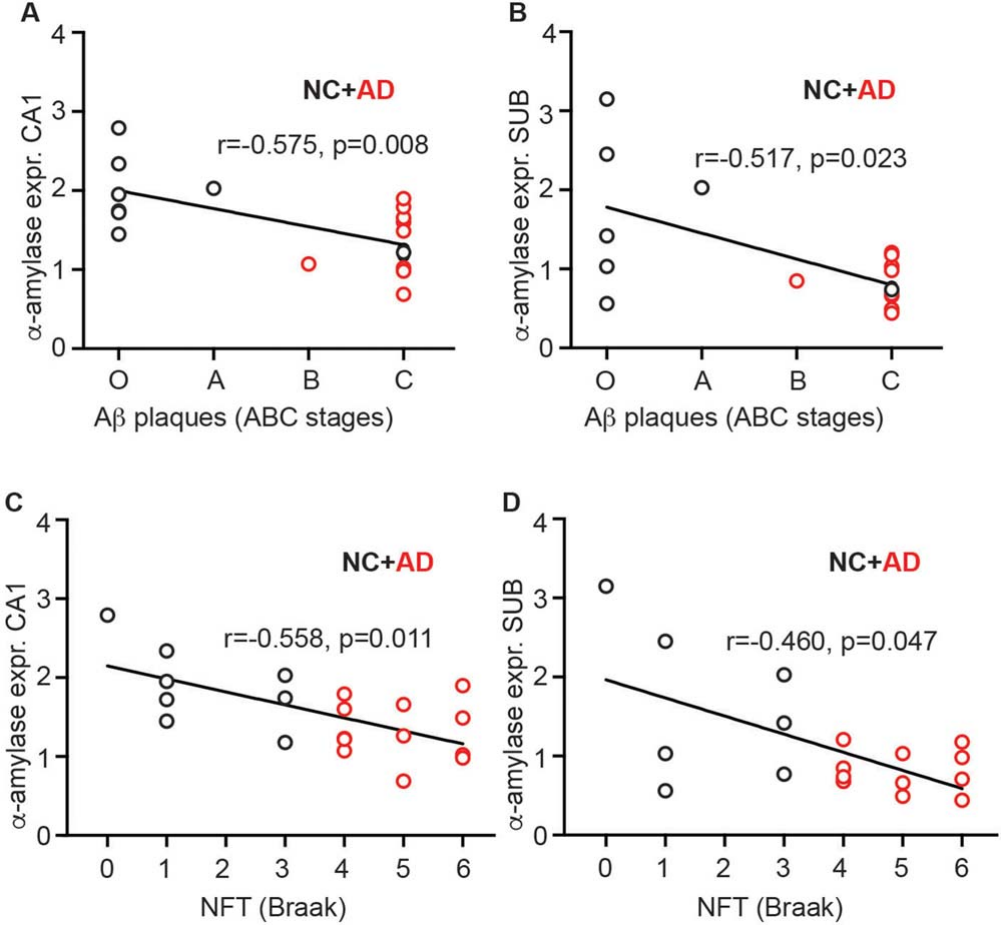
Analysis of correlations between relative alpha (α)‐amylase expression values and ABC stages of amyloid beta (Aβ) or Braak stages of NFT. Scatter plots in (**A**) and (**B**) show the negative correlation between ABC stages of Aβ plaques and the relative expression of α‐amylase in cornus ammonis (CA1) or subiculum (SUB), respectively. Scatter plots in (**C**) and (**D**) show the negative correlation between Braak stages of NFT and the relative expression of α‐amylase in cornus ammonis (CA1) or subiculum (SUB), respectively. The analyses were performed using Spearman correlation test. *indicates *P <* 0.05 ** indicates *P <* 0.01.

To further investigate the association between AD pathology and α‐amylase expression, we performed a correlation analysis between neuropathological assessment and values obtained in the RT‐qPCR analysis. We found that α‐amylase expression in both the CA1 region and the SUB region significantly and negatively correlated with ABC stages of amyloid (Figure 3A and B, respectively) and Braak stages of NFT (Figure 3C and D, respectively). We also wanted to investigate the association between Aβ levels and α‐amylase expression, we thus measured levels of Aβ in homogenates of the intermediate area between CA1 and SUB (CA/SUB). As expected, we found significantly increased levels of Aβ42 and Aβ40 in patients with AD compared with NC (53.3 ± 45.7 vs. 6.4 ± 6.3, *P =* 0.004 and 79.4 ± 55.8 vs. 30.1 ± 26.2, *P =* 0.036, respectively). Further, the Aβ42/Aβ40 ratio was increased in AD patients compared with NC (0.63 ± 0.28 vs 0.20 ± 0.16, P = 0.001), reflecting the characteristic shift towards higher Aβ42 production compared with Aβ40 production in AD patients. Correlation analysis revealed a negative and significant correlation between levels of Aβ42 and α‐amylase expression in SUB (*r* =−0.511, *P =* 0.030) as well as a trend towards a negative correlation between α‐amylase expression in SUB and Aβ42/Aβ40 ratio (*r =* −0.434, *P =* 0.072). None of the correlations were significant when NC and AD were analyzed separately and no correlations were found between α‐amylase expression in CA1 and Aβ42 levels as well as Aβ42/Aβ40 ratio regardless of whether analysis was performed across the groups or within the groups.

### Levels of alpha‐amylase are increased in Alzheimer's disease patients

To further verify the results found in our RT‐qPCR and immunohistochemical analysis, ELISA technique was used to measure the total protein levels of α‐amylase in fresh frozen brain homogenates of the CA1/SUB region from patients in cohort 2. In contrast to the RT‐qPCR finding, we found significantly higher α‐amylase concentrations in patients diagnosed with AD compared with NC (Figure 2A). However, when the analyses of AD and NC were performed separately, we observed a trend to a positive correlation between α‐amylase concentrations and SUB α‐amylase expression in AD patients (*r =* 0.503 *P =* 0.095) as well as a trend to a negative correlation between α‐amylase concentrations and CA1 α‐amylase expression in NC (*r* = −0.667 *P =* 0.071). The levels of α‐amylase were, opposed to the α‐amylase expression, positively associated with ABC stages of amyloid (*r =* 0.540, *P =* 0.014) and Braak stages of NFT (*r =* 0.482, *P =* 0.032). Increased α‐amylase concentrations were also associated with increased Aβ42/Aβ40 ratio (*r =* 0.616, *P =* 0.005), Aβ42 (*r =* 0.640, *P =* 0.003) and Aβ40 levels (*r =* 0.461, *P =* 0.047). The correlations between α‐amylase concentrations and AD pathology stages and Aβ levels did not remain significant when AD and NC groups were analyzed separately.

### Alpha‐amylase activity is increased in Alzheimer's disease patients

**Figure 4:**
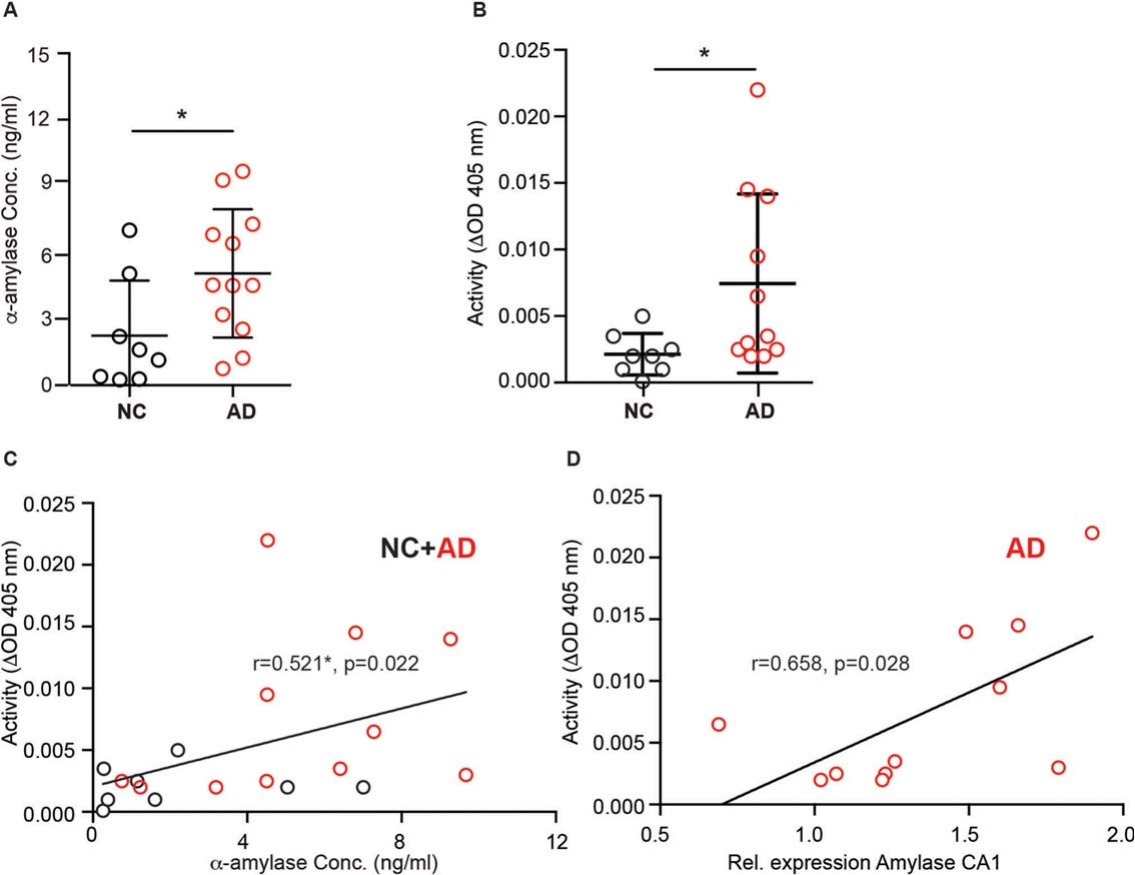
Alpha (α)‐amylase concentrations and α‐amylase activity in cornus ammonis 1 (CA1) of NC and AD patients. Scatter plot in (**A**) shows significant higher concentrations of α‐amylase in AD compared with NC. Scatter plot in (**B**) shows significant higher α‐amylase activity (optical density (ΔOD) at 405 nm during 30 minutes) in AD compared with NC. Analysis of correlations between α‐amylase activity and α‐amylase concentration shown in (**C**) demonstrates a significant positive correlation between these two variables. Scatter plot in (**D**) demonstrates the positive and significant correlation between α‐amylase activity and expression of α‐amylase in CA1 in the AD group. Comparison analyses are performed using student *t*‐test and non‐parametric Mann–Whitney. The correlation analyses were performed using Spearman correlation test. *indicates *P <* 0.05.

To further evaluate the activity of α‐amylase in brain from NC and AD patients, we performed an amylase activity assay on homogenates from hippocampus of each individual. Similar to the protein levels, the activity was significantly higher in brains from AD patients compared with NC (0.075 ± 0.0067 vs. 0.002 ± 0.0016, *P =* 0.012) (Figure 4B). Correlation analysis of α‐amylase protein levels and α‐amylase activity showed a positive and significant correlation when the analysis was performed across the groups (Figure 4C), but only a trend to significance in AD (*r =* 0.594, *P =* 0.054) and no correlation in NC group, when the groups were analyzed separately. Further, correlation analyses across the groups showed no significance between α‐amylase activity and α‐amylase expression levels in neither CA1 nor SUB. However, when the CA1 area in AD and NC group were analyzed separately, a positive correlation was found within the AD group (Figure 4D) and a trend to positive correlation within the NC group (*r =* 0.675, *P =* 0.066). Moreover, increased activity of α‐amylase was also associated with increased Aβ42 levels (*r =* 0.499, *P =* 0.035) Aβ40 levels (*r =* 0.547, *P =* 0.019) and Braak stages of NFT (*r =* 0.541, *P =* 0.017), but the correlations did not remain significant when the AD and NC groups were analyzed separately.

### Polyglucosan bodies in non‐demented controls and Alzheimer's disease patients

**Figure 5:**
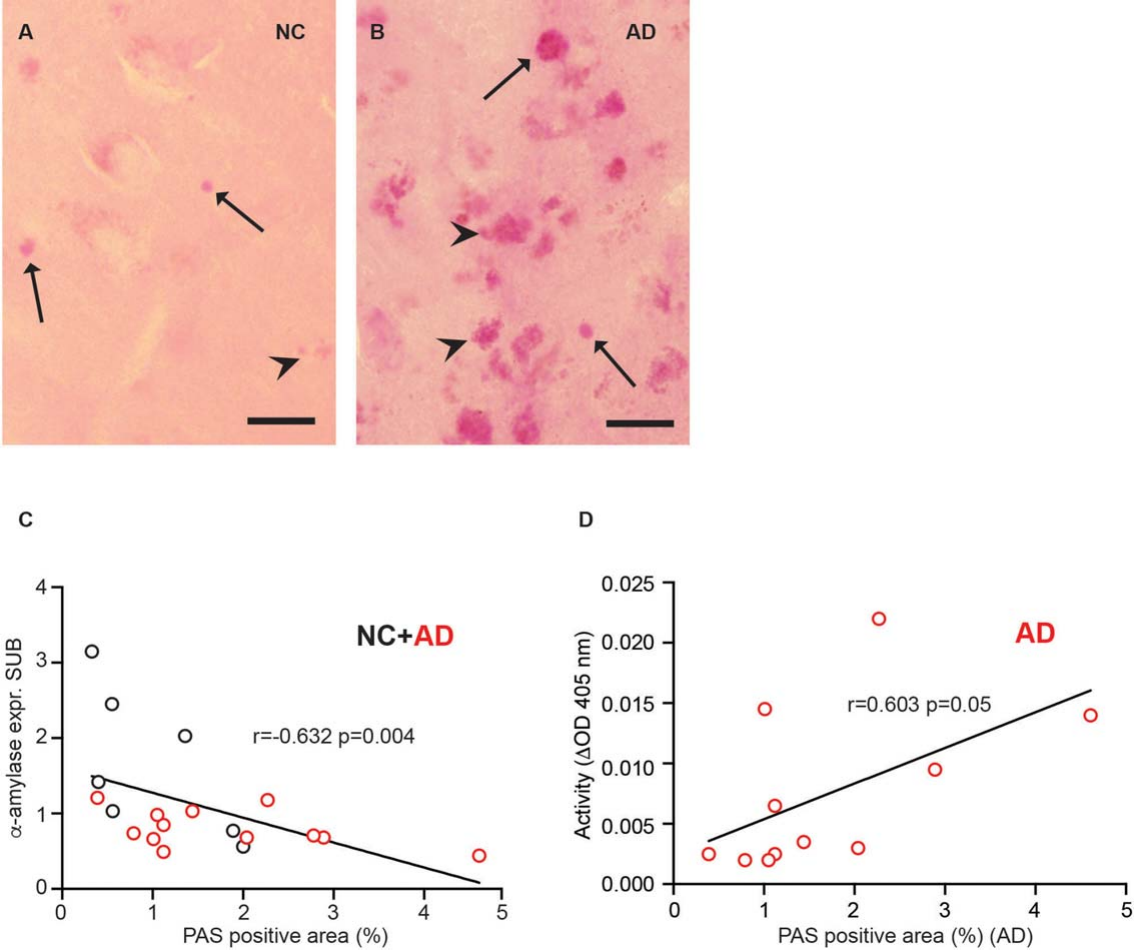
Analysis of PAS staining of brain samples from NC and AD patients. Image in (**A**) shows a PAS stained NC brain in CA1 brain area with low glycogen load, and image in (**B**) shows a PAS stain in CA1 brain area in AD brain with high glycogen load. Arrows and arrowheads (A, B) indicate round regular shaped CA inclusions and arrowheads indicate irregular shaped granular glycogen, respectively. Scatter plot in (**C**) demonstrates the correlation between alpha (α)‐amylase expression in SUB and PAS positive area in CA1/SUB. Scatter plot in (**D**) shows the correlation between α‐amylase activity [optical density at 405 nm during 30 minutes (ΔOD 405 nm)] and PAS stained area in AD patients. *indicates *P <* 0.05, **indicates *P <* 0.01. Scale bar in (A, B) = 25 µm.

Finally, we found it interesting to investigate if α‐amylase levels are related to accumulation of PGB. PAS staining of CA1 and SUB (cohort 1 and 2) revealed PGB displaying either round dense shapes (indicated with arrows in Figure 5A and B), corresponding to previously described CA 3, or irregular shapes [similar to glycogen/polyglucosan granules 19] (indicated with arrowheads in Figure 5A and B) in the CA1/SUB region. The PGBs were found in both NC and AD patients, and the AD patient with TLE displayed particular high load of the inclusions (Figure 5A–B). The percentage of PAS positive area in cohort 2 was calculated and comparative analysis showed larger, albeit not significant, areas in AD patients compared with NC (1.79 ± 1.19 vs. 1.16 ± 0.79, *P =* 0.152). To further investigate if the amount of PGBs is related to the amount of α‐amylase, we analyzed the correlation between PAS positive area and α‐amylase expression. Indeed, we found a significant negative correlation between α‐amylase expression in SUB region (Figure 5C), but when PMD was controlled for, only a trend towards correlation remained (*r* = −0.396, *P =* 0.104). No correlation between the two variables was detected in the CA1 region. Further, correlation analysis between α‐amylase activity and PAS positive area revealed a trend towards significance across the groups (*r =* 0.390, *P =* 0.099) and a significant correlation within the AD group (Figure 5D), but the correlations were lost when controlling for PMD.

## Discussion

In the current study, we use four different methods (RT‐qPCR, immunohistochemistry, ELISA and activity assay) to demonstrate the expression and presence of α‐amylase in the human brain. This novel finding has to our knowledge never been presented before, but given the fact that α‐amylase can be found in several peripheral organs, it is not unlikely that it is also expressed in the brain. Interestingly, human α‐amylase exists in various isotypes and the expression of these isotypes is organ‐specific. For example, the salivary contains AMY1A, 1B and 1C, whereas the pancreatic juice contains AMY2A and 2B 18. The heart, ovaries and thyroid glands instead contain both salivary and pancreatic isoforms 46. Our RT‐qPCR analysis does not reveal whether the brain contains several different α‐amylase (since the used primer binds to cDNA encoding several human α‐amylase isotypes including AMY1A, AMY2A, AMY2B, AMY1B and AMY1C), but the immunohistochemistry staining as well as our ELISA analysis suggest that at least some part of the enzyme pool consists of the AMY1A and AMY2A isotypes.

The presence of AMY1A in dendritic spines is intriguing as it suggests that the enzyme plays a particular role in synapse activity and plasticity. The dendrites and their spines are the most energy requiring cell components in the brain 2 and as much as 84% of the energy used during action potential transmission is consumed by postsynaptic actions 2. Recently, it has been suggested that neurons receive the majority of their energy from astrocytes and it has been claimed that neurons do not themselves have the capacity to produce, store and use glycogen. However, a number of studies, both past and recent, show that neurons do have this capacity 13, 36. Electron microscopy (EM) studies have convincingly shown glycogen grains in synapses 9 and neuronal expression of both glycogen synthases 23, 33, 40 and cytosolic GP/GDE has been described 33, 36. Thus, it is plausible that neurons, particularly at the high energy demanding post synaptic site, use glycogen as a direct energy reserve. But a high demand for energy requires a fast and efficient liberation of glucose molecules from the multi‐branched glycogen. As mentioned, GP can only degrade glycogen at the endpoints 1, which is a rate limiting factor. In contrast, α‐amylase is both very efficient (as it cleaves 5–8 glucose bonds in one enzyme–substrate interaction) and has the capability to cleave within the 1, 2, 3, 4 bond linear chain 7. In view of this capacity, we hypothesize that α‐amylase is required in post synapses in order to provide fast and continuous glucose access. Reduced amount of synaptic α‐amylase, such as the one we observed in AD patients, could thus lead to deleterious consequences from a synapse perspective.

Our study further showed that AMY1A + HB‐like inclusions were particularly found in CA1 of AD patients. HB are membrane‐free cytoplasmic inclusions containing actin and actin associated proteins as well as tau 15 and C‐terminal of the amyloid beta precursor protein (APP) 32. These inclusions can be found in elderly but are mostly associated with neurodegenerative disorders including AD 21. Its pathophysiological role is still not completely understood, but studies suggest HB as a type of aggresome formed in response to accumulation of misfolded proteins or inhibition of proteasomes 37. HB can only definitely be identified by electron microscopy (EM) 21, but (i) the found inclusions closely resembled previously published images of HB 39, (ii) the inclusions, just like HB, were primarily found in the CA1 of AD patients 37 and (iii) the inclusions co‐localized to some extent with p‐tau positive tangles, which is characteristic for HB 15. We therefore find it very likely that the inclusions are indeed HB. The co‐localization between p‐tau positive tangles and the close association with Aβ plaques moreover support the previous association between HB formation in AD pathology.

The staining against AMY1A also showed the presence of α‐amylase in pericytes. These cells play a crucial role in blood brain barrier maintenance and serve as vessel‐supporting cells. In addition, pericytes constantly surveil blood flow by regulating dilution and constriction of capillaries and this highly dynamic property requires energy. Importantly, if energy supply is lost, pericytes die and constrict the capillaries in rigor, which has detrimental impact on surrounding glial cells and neurons 20. A constant access of energy is thus of importance, which in theory could be secured by glycogen storage. Indeed, studies have shown glycogen grains in pericytes 12. Hence this cell type also needs efficient glycogen degrading enzymes such as α‐amylase.

Staining against the AMY2A isotype of α‐amylase further showed the presence of α‐amylase in astrocytes. This glial cell type is known to express both glycogen synthases and glycogen degrading enzymes such as GP, GDE and GAA 1, 11. By converting the glycogen into glucose and lactate, astrocytes are able to export an energy source which can easily be taken up at the synapse level 42. The downside of the capability of producing high amount of glycogen is reflected in the frequent presence of PGBs, particularly CA, in astrocytic processes 13. Both the neuronal energy support and its tendency to form PGBs increase the demand for efficient glycogen degrading enzymes. We observed AMY2A immunoreactivity in astrocytes in both AD and NC patients, but in AD patients these cells occasionally enclosed Aβ plaques. The presence of activated astrocytes, indicated by altered morphology and increased expression of GFAP, adjacent to Aβ plaques has been reported repeatedly and it is known that the activated astrocytes are involved in the degradation of Aβ 47. In view of these findings, it is tempting to speculate that the increased α‐amylase activity in astrocytes is required in order to either rescue dying neurons by release glucose/lactate or to increase the access of energy to be able to engulf and degrade Aβ. Support for this hypothesis can be found in a study demonstrating decreased neuronal glucose metabolism along with increased LDH activity (which may indicate increased lactate production) and astrocytic GFAP expression in the AD brain 6. Similarly, we observed low AMY1A immunoreactivity in neuronal DS in AD patients and strong immunoreactivity of AMY2A in astrocytes adjacent to Aβ plaques. In addition, we found particularly high immunoreactivity of AMY2A in astrocytes in the AD patient with TLE, a disorder associated with high neuronal energy demand, high astrocytic glycogen accumulation and high astrocytic lactate release 48.

Our immunohistochemical studies indicated alterations of α‐amylase in AD patients (fewer AMY1A + DS, increased number of AMY1A + HB and AMY2A + astrocytes gathered around Aβ plaques) and to investigate whether these alterations are reflected on a gene level, we analyzed the α‐amylase expression. We found significantly reduced expression of α‐amylase in AD patients, which inversely correlated with Aβ plaque scores, NFT scores and Aβ42 levels. These findings suggest a close relationship between α‐amylase gene expression and AD pathology and could in part explain the reduced AMY1A immunoreactivity of DS in AD patients. However, it is important to remember that only 40% of protein levels can be explained by gene expression. The remaining 60% is regulated by translational and post translational regulation as well as the ubiquitin proteasome system 43. Moreover, metabolic enzymes are particularly stable, that is, low gene expression can still yield high protein levels 43. For these reasons, it is important to also investigate potential AD‐related alterations on a protein level. Interestingly, our ELISA analysis showed, quite contrary to the RT‐qPCR analysis, increased levels of α‐amylase in AD patients compared with NC. This contradicting finding may be a result of an altered, potentially compensational, post translational regulation leading to increased α‐amylase production. Such a hypothesis is supported by findings showing repressed α‐amylase translation in response to glucose in drosophila 4 and repression of α‐amylase gene in diabetic mice with high blood glucose 26. Hence, it is possible that the reduced glucose utilization found in AD patients de‐repress translation causing elevated levels of α‐amylase. Another explanation to the elevated α‐amylase levels in AD patients might be the increased amount of AMY1A + HB in these patients. Recall that HB is believed to be formed in order to encapsulate molecule “waste,” we therefore find it possible that α‐amylase HB accumulation is a slow event proceeding along with the progressive loss of enzyme production. Since the ELISA assay measures the total concentration of both AMY1A and AMY2A present in the tissue, it cannot discriminate between potentially accumulated AMY1A in HB or AMY1A produced in DS and AMY2A in astrocytes. Nevertheless, given the found trend towards positive correlation between α‐amylase levels and α‐amylase expression in AD patients (where high numbers of HBs are present), as well as the trend towards negative correlation in NC (where none or less HBs are present), we hypothesize that increased levels of α‐amylase found in AD patients are in part due to increased accumulated α‐amylase in HB aggresomes.

Activity of enzymes does not always coincide with protein levels. However, in contrast to many enzymes (which often require conversion from proactive to active forms in order to yield their enzymatic properties) α‐amylase becomes activated when polysaccharides bind to the active site of the enzyme and thus usually α‐amylase protein levels and α‐amylase activity correlate very well. Our activity analysis showed that α‐amylase activity is, in concert with the protein levels of α‐amylase, increased, but this positive correlation was only seen when the analysis was performed across the groups and not when the AD and NC groups were analyzed separately (only a trend remained). The lack of complete correlation can possibly be explained by methodological issues. The ELISA used for the α‐amylase levels detects both AMY1A and AMY2A, but how much of each isoform the kit detects is difficult to specify. The activity assay on the other hand captures the activity of all α‐amylases in the brain. Moreover, the two methods require different pretreatment of the tissue (which can affect the outcome of the α‐amylase values) and consequently the analysis is performed on different tissue samples from the same individual. These obstacles can all contribute to the discrepancy between the amount of α‐amylases measured by the two methods, however the fact that we do find a correlation (despite the obstacles) indicate that the results obtained by the methods are valid for interpretation.

In addition, although gene expression decreased and activity increased in AD patients, we found a positive correlation between the two variables when AD and NC were analyzed separately. Hence, it appears as if both expression of α‐amylase and translational regulation determine the protein and activity levels.

To finally investigate the potential consequences of altered α‐amylase gene expression, levels and activity in AD patients, we investigated the link between α‐amylase and PGBs. As mentioned previously, several studies have demonstrated increased amount of CA and PGBs in AD patients [for review see 35] and our own study showed increased, albeit not significant, PGB load in AD compared with NC brains. The lack of significance could be explained by the fact that PGB is associated by PMD 23, something we also noted in our own study. Interestingly, we found a strong negative correlation between α‐amylase expression in SUB and PAS positive area. The significance of the correlation was lost after controlling for PMD, but the finding still indicates a link between α‐amylase and PGB formation. We also found increased α‐amylase activity in individuals with high PGB load, a correlation reflecting the increased amounts of polysaccharides binding to activation sites of the enzyme in AD patients. Interestingly, increased PGB load in conjunction with elevated levels of glycogen degrading enzymes, such as GP, has been reported before 23, which suggests that increased activity of degrading enzymes is a general compensatory event evoked in response to PGB formation.

To conclude, our studies show that α‐amylase is present in the human brain and that the gene expression, concentration and activity of the enzyme are altered in the AD brain. Given that the primary function of α‐amylase in the periphery is to degrade polysaccharides and thereby release glucose, we hypothesize that the enzyme plays a similar role in the brain. We therefore propose α‐amylase as a novel actor potentially involved in the alterations of glucose availability and neuropathological changes observed in patients with AD.

## Conflict of Interest

The authors declare that they have no competing interest.

## Author Contributions

EB designed the study, carried out the RT‐qPCR analysis, α‐amylase activity, ELISA assay, immunohistochemical staining and analyzed the data. NS performed immunohistochemical staining and contributed with manuscript preparation. NBB collected the brain tissue samples and performed the neuropathological evaluation. MF supervised the RT‐qPCR analysis. MW coordinated the study, performed immunohistochemical staining and edited the manuscript. All authors read and approved the final manuscript.

## Supporting information


**Figure S1.** Immunohistochemical stainings of NC and AD patient hippocampal CA1 using antibodies directed against the full‐length native human salivary α‐amylase (nAMY1A) (**A–C**) and full‐length native human pancreatic α‐amylase (nAMY2A) (**C–E**) made in sheep (Abcam). As demonstrated in image (A) the nAMY1A antibody stained dendritic like projections (indicated with arrows in A) in NC, whereas the staining of CA1 from an AD patient (indicated with arrows in B) revealed HB‐like inclusions, less dendritic staining and pericyte like cell bodies (indicated with arrows in C). Scale bar = 10 μm. Image in (D) show a western blot membrane stained with AMY1A. Wells loaded with hippocampal NC homogenates and standard human salivary α‐amylase (separated by a protein ladder) showed bands at approximately 50–52 kDa. Two additional band around 40 kDa appeared in the well loaded with homogenates. The staining against nAMY2A (**E–G**) stained foremost glial cells. The glial cells were weakly stained in NC (indicated with arrows in E), but scattered glial cells were strongly stained in AD (indicated with arrows in F) patients. The patients with AD patients and TLE showed an overall strong AMY2A immunoreactivity (indicated with arrows in G). Scale bar = 10 μm. Image in (**H**) shows a western blot membrane stained against AMY2A. Wells loaded with pancreatic porcine α‐amylase and hippocampal AD homogenates showed bands at 50–52 kDa. Two additional bands, located between 40 and 50 kDa, were detected in the latter well.
