# Correction to “Brain alpha‐amylase: A novel energy regulator important in Alzheimer disease?”

**DOI:** 10.1111/bpa.70055

**Published:** 2025-11-26

Byman E, Schultz N, Netherlands Brain Bank, Fex M, Wennström M. Brain alpha‐amylase: a novel energy regulator important in Alzheimer disease? *Brain Pathology*.2018;28: 920–932. https://doi.org/10.1111/bpa.12597

In this article, the incorrect version of image **H** in **Figure 1** was published. The image depicts amylase staining of a pericyte/vessel. Certain red areas adjacent to the pericyte and vessel had been removed. This altered image was accidentally included in the publication.

The corrected version of **Figure 1** is provided below with the original image restored in panel **H**.

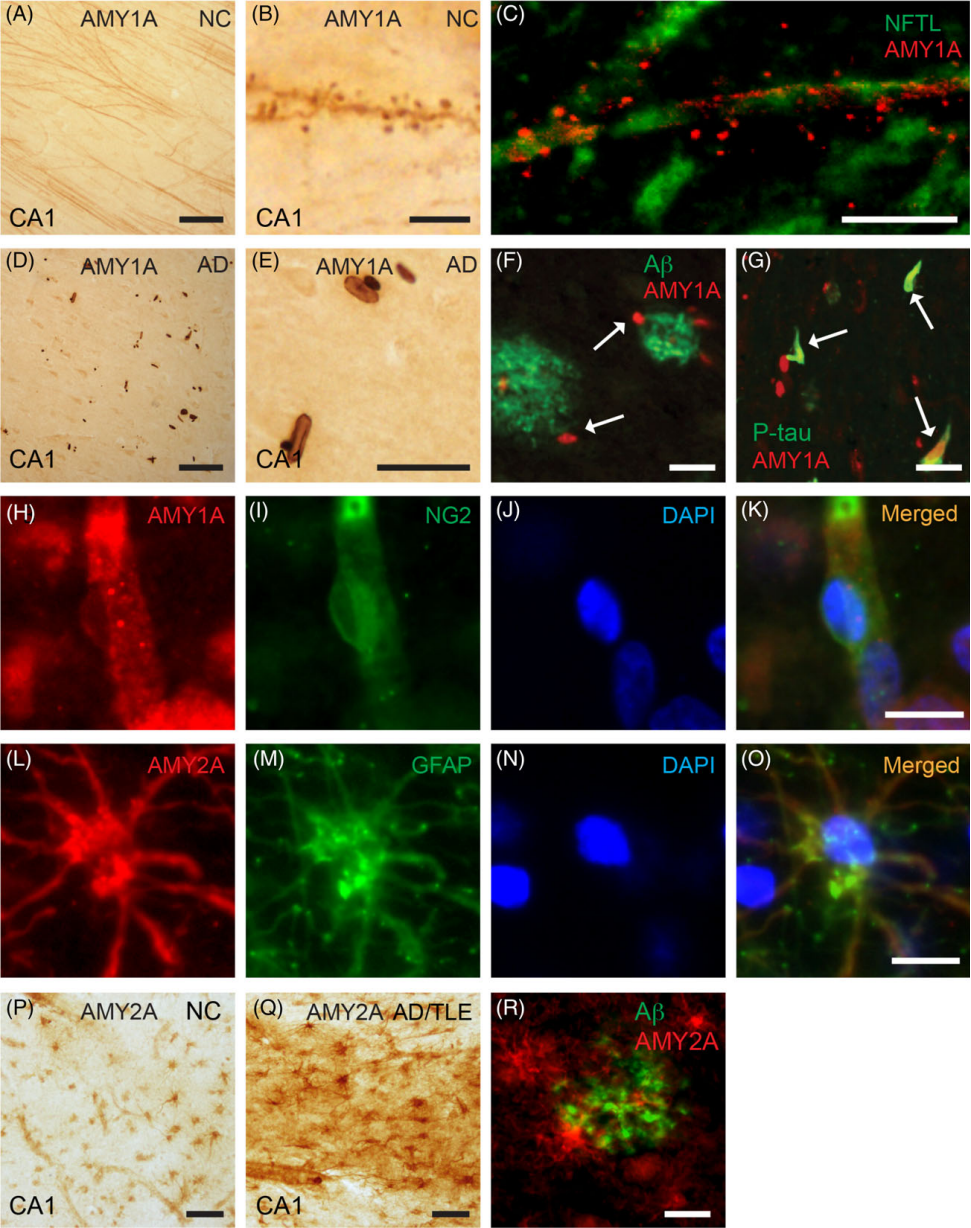

This correction does not affect the results, interpretations, or conclusions of the study, as the main findings concern neurons rather than pericytes.

We apologize for this error.

