# Supplementary figures and images for: Brain alpha‐amylase: a novel energy regulator important in Alzheimer disease?
Source: Brain Pathol. 2018 Mar 30;28(6):920–32. doi: 10.1111/bpa.12597 (PMC8028266; doi:10.1111/bpa.12597)

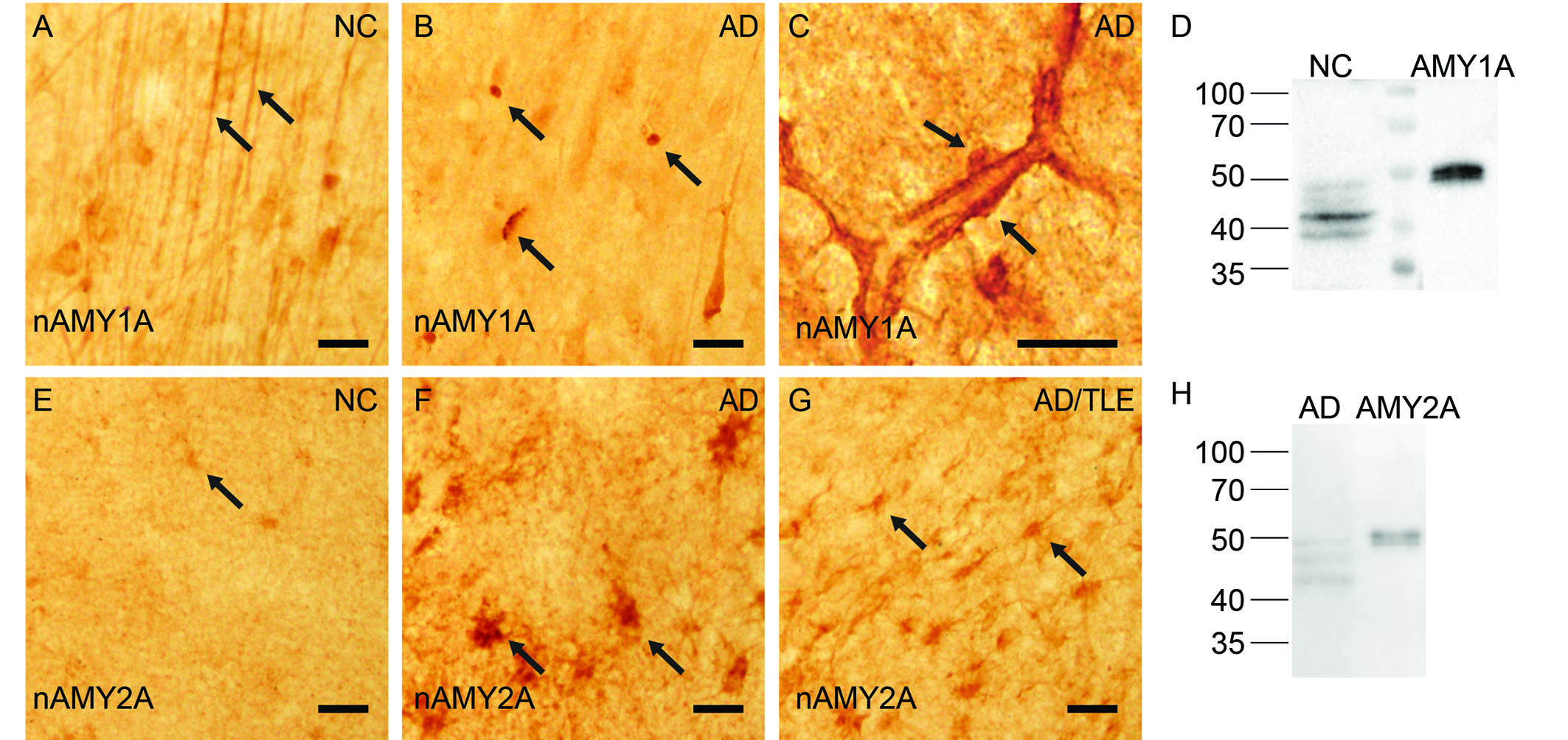

Supplement: Supplementary file 1 — Figure S1. Immunohistochemical stainings of NC and AD patient hippocampal CA1 using antibodies directed against the full‐length native human salivary α‐amylase (nAMY1A) (A–C) and full‐length native human pancreatic α‐amylase (nAMY2A) (C–E) made in sheep (Abcam). As demonstrated in image (A) the nAMY1A antibody stained dendritic like projections (indicated with arrows in A) in NC, whereas the staining of CA1 from an AD patient (indicated with arrows in B) revealed HB‐like inclusions, less dendritic staining and pericyte like cell bodies (indicated with arrows in C). Scale bar = 10 μm. Image in (D) show a western blot membrane stained with AMY1A. Wells loaded with hippocampal NC homogenates and standard human salivary α‐amylase (separated by a protein ladder) showed bands at approximately 50–52 kDa. Two additional band around 40 kDa appeared in the well loaded with homogenates. The staining against nAMY2A (E–G) stained foremost glial cells. The glial cells were weakly stained in NC (indicated with arrows in E), but scattered glial cells were strongly stained in AD (indicated with arrows in F) patients. The patients with AD patients and TLE showed an overall strong AMY2A immunoreactivity (indicated with arrows in G). Scale bar = 10 μm. Image in (H) shows a western blot membrane stained against AMY2A. Wells loaded with pancreatic porcine α‐amylase and hippocampal AD homogenates showed bands at 50–52 kDa. Two additional bands, located between 40 and 50 kDa, were detected in the latter well. [file BPA-28-920-s001.jpg]
